# The MindGomoku: An Online P300 BCI Game Based on Bayesian Deep Learning

**DOI:** 10.3390/s21051613

**Published:** 2021-02-25

**Authors:** Man Li, Feng Li, Jiahui Pan, Dengyong Zhang, Suna Zhao, Jingcong Li, Fei Wang

**Affiliations:** 1 School of Computer and Communication Engineering, Changsha University of Science and Technology, Changsha 410114, China; limancsust@gmail.com (M.L.); Lif@csust.edu.cn (F.L.); zhdy@csust.edu.cn (D.Z.); 2 Hunan Provincial Key Laboratory of Intelligent Processing of Big Data on Transportation, Changsha University of Science and Technology, Changsha 410114, China; 3 School of Software, South China Normal University, Guangzhou 510631, China; panjiahui@m.scnu.edu.cn (J.P.); lijingcong@hotmail.com (J.L.); 4 Pazhou Lab, Guangzhou 510330, China; 5 College of Electrical and Information Engineering, Zhengzhou University of Light Industry, Zhengzhou 450002, China; zsnzsn1221@163.com

**Keywords:** brain–computer interface (BCI), electroencephalogram (EEG), P300, BCI game, Bayesian deep learning

## Abstract

In addition to helping develop products that aid the disabled, brain–computer interface (BCI) technology can also become a modality of entertainment for all people. However, most BCI games cannot be widely promoted due to the poor control performance or because they easily cause fatigue. In this paper, we propose a P300 brain–computer-interface game (MindGomoku) to explore a feasible and natural way to play games by using electroencephalogram (EEG) signals in a practical environment. The novelty of this research is reflected in integrating the characteristics of game rules and the BCI system when designing BCI games and paradigms. Moreover, a simplified Bayesian convolutional neural network (SBCNN) algorithm is introduced to achieve high accuracy on limited training samples. To prove the reliability of the proposed algorithm and system control, 10 subjects were selected to participate in two online control experiments. The experimental results showed that all subjects successfully completed the game control with an average accuracy of 90.7% and played the MindGomoku an average of more than 11 min. These findings fully demonstrate the stability and effectiveness of the proposed system. This BCI system not only provides a form of entertainment for users, particularly the disabled, but also provides more possibilities for games.

## 1. Introduction

The brain–computer interface (BCI) is an unconventional communication method that builds a communication path between the human and the peripheral without any muscular activities [1,2]. Motivated by the demands of people with physical disabilities, researchers initially applied BCI technology to the clinical field to help patients regain their ability to interact with the outside world by sending commands directly from the brain to the computer [3,4]. Apart from clinical applications, BCI technology has also been experimented with and applied in entertainment games [5,6]. Over the past decade, researchers have developed several BCI-based video games, in which BCI technology is generally used to provide input for games so as to get rid of complete dependence on intermediate devices (mouse, keyboard, gamepad, and game controllers) [7]. BCI games constitute a platform that may satisfy the interests of both healthy users and disabled users. For healthy users, BCI games are mysterious and technical, which increases the charm of the games and is very conducive to the promotion of the games. For users with disabilities, BCI games provide them with a fair gaming platform that not only allows them to play games with healthy users in the same way but also can be used as a functional rehabilitation system to help patients with rehabilitation training [8]. Furthermore, gaming can be an excellent motivation to spend time with a BCI system in order to achieve better control [9], and applying BCI technology to entertainment games is an important link to promote BCI technology from the scientific research stage to the practical application market stage [10].

In recent years, the application of BCI technology to game interaction has become increasingly popular, and plenty of research has been done in the past decade [11]. Among the BCI games in previous studies, P300 potential, steady-state visually evoked potentials (SSVEPs), and motor imagery (MI) are the most common games using electroencephalogram (EEG) signals [12]. Table 1 summarizes related works on BCI games. Martinez et al. presented a new SSVEP-based BCI system that allows a BCI user to navigate a small car on the computer screen in real time, in any of the four directions, and to stop it, if necessary. Finally, the average online accuracy of the low-frequency (LF) range (5, 6, 7, 8 Hz) reached 93%, and the average online accuracy of the intermediate-frequency (MF) range (12, 13.3, 15, 17 Hz) reached 96.5% [13]. Bonnet et al. created a multiuser videogame called BrainArena, in which two users can play a simple football game by means of two BCIs. The mean classification accuracy in single-player mode was 71.25% and that in two-player mode 73.9% [14]. Martišius et al. developed an online shooting game based on the SSVEP paradigm using the Emotiv EPOC headset. The system uses wave atom transform for feature extraction, achieving an average accuracy of 78.2% using a linear discriminant analysis classifier, 79.3% using a support vector machine classifier with a linear kernel, and 80.5% using a support vector machine classifier with a radial basis function kernel [15]. Wang et al. combined MI and SSVEP to generate multiple commands to jointly control Tetris. The classification accuracy of MI and SSVEP reached 87.01% and 90.26, respectively [10]. Finke et al. proposed the P300-based MindGame, and the classification rate of a single trial during the online operation reached 66% [16]. Angeloni et al. proposed a memory game based on a P300 BCI and achieved an average overall accuracy of 88.47% [17]. A BCI game based on motor imagination usually has high requirements for the subjects, and it takes a training course of some duration to be well in control of the game [18]. BCI games based on SSVEP have a higher accuracy rate. However, the band of easily detectable frequencies is very limited, so the number of controls that are coded by different frequencies is also limited [7]. In addition, SSVEP-based BCIs require concentration on a stimulus flickering at a constant rate, which may cause fatigue and, in certain settings, even epileptic seizures [19]. In the commonly used paradigms, P300 is stable, is less prone to inducing fatigue, and does not require users with special training. The performance of the earlier P300 BCI game, however, has generally been poor. There is still room for improvement in the accuracy of BCI games based on P300. Therefore, in this study, we design a high-performance and stable P300 BCI game. Furthermore, previous studies have primarily focused on applying existing BCI technology to games directly, while ignoring the essence of games. Generally, a mature game requires that the operation process be as simple as possible, the user interface be simple, and the user’s commands get a quick response. In addition, the game should run normally in an uncontrolled environment and a wider user group [20]. With the aim of making BCI games more humane and user friendly, the strategy of combining control methods and games still needs to be further improved.

Most current technologies in BCIs use traditional machine learning algorithms for decoding. Numerous classification approaches have been introduced in previous studies [21,22,23,24], such as linear discriminant analysis (LDA) and support vector machine (SVM). The past decade has seen the rapid development of deep learning in many fields, such as computer vision, natural language processing, and speech processing [25]. Researchers have proposed some novel deep architectures, such as AlexNet [26], VGGNet [27], and GoogleNet [28], which have achieved high accuracy in image classification. In recent years, the convolutional neural network (CNN) has been used for P300 detection, which shows comparable performance with the traditional machine learning techniques. Cecotti and Graser took the lead in using a CNN to identify and classify P300 EEG signals, finally achieving good results [29]. Liu and Wu developed a five-layer CNN with batch normalization and dropout to improve the generalization of the model and achieved state-of-the-art P300 signal classification and character recognition [30]. As a CNN requires a large data set to train for nonlinear data, overfitting will occur on small data sets. However, it is very difficult to collect a large amount of training data in actual applications. Thus, we propose a novel architecture based on a Bayesian convolutional neural network (BCNN) to achieve state-of-the-art P300 signal classification and character recognition on a small training data set.

Previous research on P300 BCI technology mainly focused on data processing algorithms and paradigm optimization. The optimization study of the experimental paradigm is a key issue in improving the performance of BCI systems that considers both accuracy and speed [31,32,33]. Farwell and Donchin designed the first P300-based speller in 1988 and proposed a visual oddball paradigm [34]. Although this paradigm has been most commonly used and tested with various configurations, it remains subject to adjacency distraction and double-flash errors [35]. After that, researchers proposed various other paradigms. Guan proposed the single-cell paradigm (SCP), in which each character is flashed randomly and individually [36]. Fazel-Rezai proposed the region-based paradigm (RBP), in which character recognition is done at two levels [37]. In this study, we introduced a game interactive paradigm for the proposed system to improve efficiency.

In this study, we design a P300 BCI game based on a BCNN, called the MindGomoku, which is based on the classic two-player pure strategy chess game Gomoku. In the MindGomoku system, the players alternately place the Go pieces (black and white) on an empty intersection. In each turn, the player should choose one position to lay down a piece through the proposed BCI system, which can effectively prevent the player from having to continuously select the target position on the Go board and does not easily cause visual fatigue. In addition, we propose an algorithm based on Bayesian deep learning, which solves the overfitting problem when training on small data sets. The experimental results in this study will prove the successful application of not only P300-based BCI games but also the deep learning algorithm that can be applied to online BCI systems.

The contributions of our work can be summarized in four points:

(1) A new BCI video game based on P300 called the MindGomoku: We propose and implement this game.
(2) The introduction of a P300 interactive visual stimulation paradigm for BCI applications: We present a paradigm based on BCI user feedback.
(3) A novel simplified Bayesian convolutional neural network (SBCNN) architecture for P300 detection.
(4) An evaluation of our system on 10 naive subjects.

This article is organized as follows: Section 2 describes the system framework, paradigm design, data acquisition, algorithm, and game design. The experiments and results are provided in Section 3, and Section 4 provides a further discussion and explanation of the experimental results.

## 2. Methods

### 2.1. System Framework

**Figure 1:**
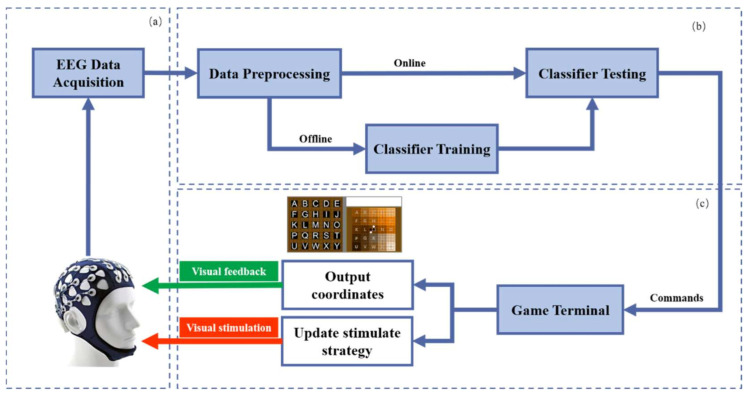
The framework of the BCI game, which contains three subsystems as follows: (**a**) data acquisition, (**b**) data processing, and (**c**) visual and game terminal. The data acquisition part records the electroencephalogram (EEG) signal. After the signal is preprocessed, the data processing part can be divided into two steps, off-line classifier training and online classifier testing. The visual and game terminal provides users visual stimuli after updating the stimulus strategy and provides corresponding visual feedback (output coordinates).

The system framework contains three subsystems, the data acquisition section, the data processing section, and the visual and game terminal, as shown in Figure 1. In the data acquisition section, multi-channel scalp EEG signals are recorded using electrode caps and amplifiers. After the signal is preprocessed, the data processing part can be divided into two steps, off-line training and online classification testing. Finally, the classification results are converted into operation commands and sent to the visual and game terminal. The details of the classifier are described in Section 2.4. The visual and gaming terminal includes two sub-steps: (1) provide visual stimulation to the user after the stimulation strategy is updated and (2) provide the user with visual feedback (output coordinates).

### 2.2. Paradigm Design

**Figure 2:**
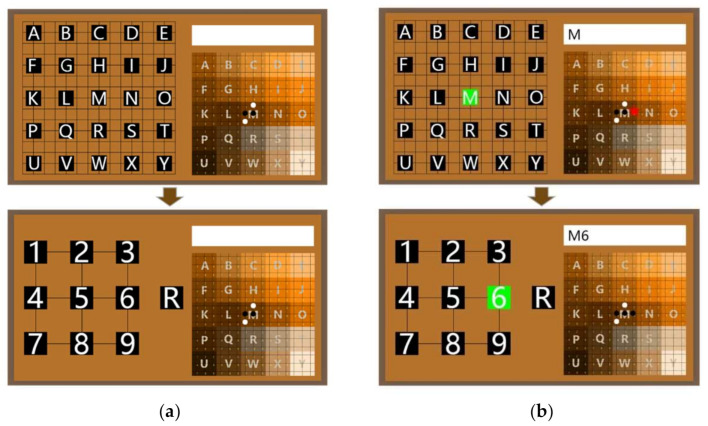
An illustration of the MindGomoku. (**a**) The graphical user interface of the MindGomoku. (**b**) The online interactive paradigm of the MindGomoku. It takes two steps to select the coordinate of the red star. According to its location, the user should select the character M in the first-level interface and then select the character 6 in the second-level interface. The two selections can determine a coordinate, at which the system will present a piece in the Go board as feedback.

The graphical user interface (GUI) of the MindGomoku is presented in Figure 2a and consists of three parts: Go board (bottom right), textbox (upper right), and visual stimulation panel (left). There are 15 × 15 points on the board, which are divided into 25 areas. The center of each area is marked with a character, which contains 9 points. The board and the textbox are used for feedback on each selection to help users determine whether they have made a wrong selection. The visual stimulation panel is used to provide stimulation by flashing different buttons, 25 buttons in the first interface and 10 buttons in the second interface.

We designed a visual stimulation paradigm for the MindGomoku game based on its own characteristics. In this paradigm, the user needs to make two choices through a dual interface for each piece played. For example, it takes two steps to select the coordinate of the red star, as shown in Figure 2b. Since it is located in the area represented by M, the first step is to select the character M in the first-level interface. The result of the first selection will be the output in the textbox, and the stimulation panel will automatically switch to the second-level interface. In the second step, the user should select character 6 in the second-level interface according to the location of the red star. The result of this selection will also be displayed in the textbox. Furthermore, the two selections can determine a coordinate, at which the system will present a piece. As in the traditional human–computer Gomoku, the MindGomoku game will automatically play with the user to achieve a man–machine battle when the user successfully selects a board coordinate to place the piece. Meanwhile, the content of the textbox will be cleared. Finally, the side that first connects five pieces will win, and the system will issue an audible congratulatory note.

In addition, we call the procedure of output of a coordinate as a trial and the required two selection processes as two sub-trials. In the first-level interface, there are 25 buttons in the visual stimulation panel, representing the 25 areas on the board. In the first sub-trial, all 25 character buttons are successively flashed randomly in each round, repeating 10 rounds. In each flash, the character button is intensified for 100 ms. The inter-stimulus interval (ISI) is 40 ms [31], and there is no time gap between successive rounds. In the second-level interface, there are 9 digital buttons to represent further locations of the piece and a character button R to give the user the regret option and allow the user to return to the first-level interface. In the second sub-trial, the system adopts an optimization paradigm called the game interactive sequence shortening (GI-SS) method. In this method, we use a dynamic flashing sequence to shorten the flashing time, which is generated by removing the valid buttons representing the occupied positions from all buttons. With the purpose of detecting the P300 potential reliably, the shortening should stop when the dynamic queue is reduced to a certain length and the minimum sequence length is set to 6 in this study. The dynamic flashing sequence is generated or updated at the beginning of the second sub-trial. The same as the first sub-trial, the buttons in the dynamic flashing sequence are successively flashed randomly in each of 10 rounds. The other parameters of the second-level interface are the same as the first-level interface.

### 2.3. EEG Data Acquisition and Preprocessing

EEG data are non-invasively recorded using 32-channel caps (Electro-Cap International, Inc., Eaton, OH, USA) and SynAmps2 amplifiers (Compumedics, Neuroscan, Inc., Charlotte, NC, USA), digitized at 1000 Hz and filtered using a 50 Hz notch filter. Data are collected from all electrodes except A1 and A2. All electrode impedances are maintained below 5 kΩ.

To reduce the influence of the filter edge effect, the recorded data are filtered first [38]. Band-pass filtering is used for the EEG signal from each channel, with a cutoff frequency of 0.1–20 Hz. We take the time window of 600 ms after a character flashing as an epoch, which is used to capture the necessary information about the P300 signal after stimulation. Then, the data from an epoch are downsampled with a rate of 4. This results in EEG data built in a matrix of 150 × 30. We superimpose and average the data matrices of the same character flashing to reduce the signal-to-noise ratio. Due to the design of the proposed paradigm, the sample sizes of P300 and non-P300 data are unbalanced. To solve this problem, we artificially copy the positive training samples to achieve the same positive and negative training samples.

### 2.4. SBCNN Architecture

After the signal is preprocessed, the data processing part can be divided into two steps, off-line training and online classification. In this paper, we propose the SBCNN algorithm. The SBCNN is based on Bayes by Backprop (BBB), which is a variational inference method to learn the posterior distribution of the weights of a neural network from which weights can be sampled in backpropagation. For example, if a Gaussian distribution is used to represent each weight parameter, the original weight value can be expressed as the parameters of the Gaussian distribution, that is, the mean and the standard deviation. Then the posterior is calculated by variational reasoning [39].

The weight $w∼p\left(w|D\right)$ of the Bayesian neural networks (BNNs) can be sampled from the weight w in the backpropagation. However, the true posterior is usually difficult to solve, so an approximate distribution $q_{\theta }\left(w|D\right)$ is defined and the approximate distribution is used to approximate the real distribution $p\left(w|D\right)$, where the specific shape is represented by parameters that can be calculated by minimizing the Kullback–Leibler (KL) divergence. The optimization parameters $\theta^{opt}$ are defined as:

(1) $$\theta^{opt}=\underset{\theta }{\arg\min}\mathrm{KL}\left[q_{\theta }\left(w|D\right)∥p\left(w|D\right)\right]\;|=\underset{\theta }{\arg\min}\mathrm{KL}\left[q_{\theta }\left(w|D\right)∥p\left(w\right)\right]-E_{q\left(w|\theta \right)}\left[\log p\left(D|w\right)\right]+\log p\left(D\right)\;|=\underset{\theta }{\arg\min}\int q_{\theta }\left(w|D\right)\log\frac{q_{\theta }\left(w|D\right)}{p\left(w\right)}dw-E_{q\left(w|\theta \right)}\left[\log p\left(D|w\right)\right]+\log p\left(D\right)$$

where the term $\log p\left(D\right)$ is constant, so it can be omitted in the optimization. The KL divergence is also intractable to compute exactly. Gal and Ghahramani used Monte Carlo integrals on network weights to approximate integrals in KL divergence, and the process of minimizing the KL divergence is equivalent to performing Monte Carlo dropout training [39].

The convolutional layer of a CNN needs to use a convolutional kernel with a fixed weight value for the convolutional operation. However, each weight parameter in the convolutional kernel of a Bayesian convolutional neural network (BCNN) is expressed in the form of a Gaussian distribution. To obtain a certain weight value, it is necessary for a Gaussian distribution to be used for sampling. However, the sampling process is not derivative in the forward propagation, and in the training process, the network cannot be trained using the backpropagation method. Therefore, in the sampling process, the reparameterization technique is used to put the sampling process in front so that the forward propagation of the network becomes derivative and the weights are updated in the backpropagation process. A certain weight value is sampled from the weight distribution of the convolutional kernel, and the weight value obtained in this way is formed into a convolution kernel, and a convolution operation is performed on the receiving field. This procedure can be defined as follows:

(2) $${\mathrm{sample}}_{\mathrm{conv}}={\mathrm{conv}}_{\mathrm{mean}}+\epsilon {\mathrm{conv}}_{\mathrm{std}}$$

(3) $$S=R*{\mathrm{sample}}_{\mathrm{conv}}$$

The reparameterization method is used in the fully connected layer, and the weight parameters are sampled. μ represents the expectation of the distribution, and σ represents the standard deviation of the distribution. ϵ is the sampling of the standard Gaussian distribution. For each weight parameter, it is defined as a function according to the following formula:

(4) $$f\left(\epsilon \right)\;=w=\mu +\epsilon *\sigma ,\epsilon \in N\left(0,1\right)$$

This parameter update procedure can be defined as follows:

(5) $$\Delta \mu =\frac{\partial f}{\partial w}+\frac{\partial f}{\partial \mu }$$

(6) $$\Delta \sigma =\frac{\partial f}{\partial w}\frac{\epsilon }{\sigma }+\frac{\partial f}{\partial \sigma }$$

(7) μ←μ−αΔμ

(8) σ←σ−αΔσ

(9) $$\theta^{*}=\left(\mu^{*},\sigma^{*}\right)$$

where $\theta^{*}$ is the updated parameter.

**Figure 3:**
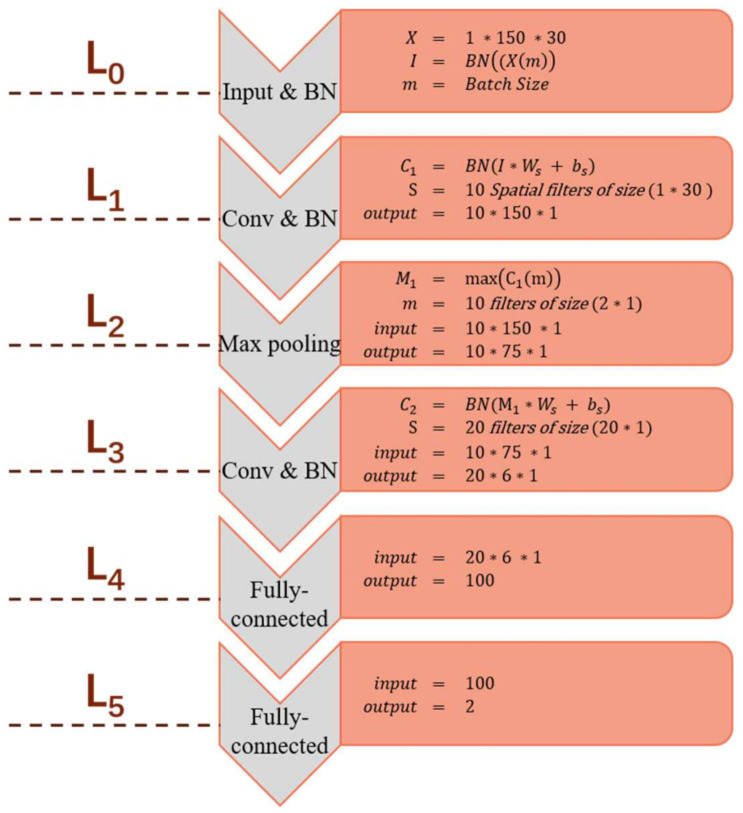
Architecture of a simplified Bayesian convolutional neural network (SBCNN) for feature extraction and classification.

In a BCNN, because the Gaussian distribution is used to make the prior probability distribution for each weight parameter, the single-point estimation in a traditional CNN is expanded into a Gaussian distribution form composed of the mean and variance. The parameters in the BCNN can be expressed as $\theta =\left\{\mu ,\sigma^{2}\right\}$. A BCNN model with a complex structure will be difficult to converge, and the generalization ability of the model will also be reduced. The SBCNN is proposed to detect P300. In this work, we implemented a six-layered SBCNN for the detection of the targeted and non-targeted P300 components shown in Figure 3. In this architecture, we introduced three batch normalization (BN) [40] layers, at L0, L1, and L3, to reduce the saturation of the gradient during covariate shift as it normalizes input features in a batch, and the rectified linear unit (ReLU) activation function is used at each convolutional layer. The network topology is described as follows:

L0 (input and BN layer): $I_{i,j}$ with $0\leq i<N_{\mathrm{elec}}$ and $0\leq j<N_{t}$. In this work, $N_{\mathrm{elec}}=30$, $N_{t}=150$, and the batch size for stochastic gradient descent is set to 85.

(10) $$I=BN\left(X\right)$$

L1 (convolution and BN layer): Spatial filters of size (1 × $N_{\mathrm{elec}}$) are convolved with the input normalized batch over the length of the signal to extract hierarchical features from the input, and the BN layer is used after the convolutional layer. The mathematical expression is as follows:

(11) $$C_{1}\left(j\right)\;=f\left(\sum_{i=1}^{N}I_{i}\left(j\right)*W_{s}^{\left(1\right)}\left(j\right)+b_{s}^{\left(1\right)}(j)\right)$$

where $C_{1}\left(j\right)$ is output data for L1, $f\left(x\right)$ is the ReLU activation function, and $W_{s}^{\left(1\right)}$ and $b_{s}^{\left(1\right)}$ are the weights and biases of the spatial filter, respectively.

L2: The pooling layer consists of a pooling filter of size (2 × 1) and a stride size of 2. The output of this layer is defined as $M_{1}$.

L3: (convolution and BN layer): Temporal filters of size (1 × 20) are used to extract temporal features. The mathematical expression is as follows:

(12) $$C_{2}\left(j\right)\;=f(BN(\sum_{s=1}^{s\leq N_{s}}\sum_{t=1}^{t\leq N_{t}}(M_{1}\left(t\cdot 20+j\right)\cdot W_{s}^{\left(2\right)}\left(j\right)+b_{s}^{\left(2\right)}\left(j\right))))$$

where $C_{2}\left(j\right)$ is the output data for L3, $N_{s}=10$, $f\left(x\right)$ is the ReLU activation function, and $W_{s}^{\left(2\right)}$ and $b_{s}^{\left(2\right)}$ are the weights and biases of the filter, respectively.

L4 and L5: These two layers are fully connected layers. The calculation formula is as follows:

(13) $$F_{1}\left(j\right)\;=\sigma (\sum_{i=1}^{i\leq 100}(C_{2}\left(j\right)\cdot w^{\left(1\right)}+b^{\left(1\right)}))$$

(14) $$F_{2}\left(j\right)\;=\overset{i\leq 2}{\underset{i=1}{\sum }}(F_{1}\left(j\right)\cdot w^{\left(2\right)}+b^{\left(2\right)})$$

where $F_{1}\left(j\right)$ is the output data for L4, $\sigma \left(x\right)$ is the Softmax function, and w and *b* are the weights and biases, respectively, from which the location of the maximum probability value can be selected to determine the target character. The formula is as follows:

(15) $$R=\arg_{i}\max p\left(K,i\right)\;\;\left(1\leq i\leq C\right)$$

where R represents the final selected target, p denotes the probability value, i denotes the position number, and C denotes the number of optional characters.

The recorded EEG signals are input into the proposed SBCNN algorithm after preprocessing. Let X denote the N × 150 × 30 input tensor and N denote the number of target characters in the paradigm we proposed (in the first sub-trial, N = 25, and in the second sub-trial, N depends on the actual situation of the game (6 ≤ N ≤ 10)). After normalizing the input with batch normalization, spatial filtering is used to extract spatial features and ReLU is used to perform nonlinear mapping on the output of the convolutional layer. Then, after pooling the spatial feature map, the temporal filter is used to extract the temporal features and ReLU is used to perform nonlinear mapping. The fully connected layer is used for classification to get the probability of P300 and non-P300. Finally, we take the largest P300 probability corresponding to N characters as the prediction result output. The algorithm process is summarized in Algorithm 1.
**Algorithm 1** The proposed character recognition algorithm**Input:** EEG Data, X with the size of (N × 150 × 30)**Output:** Predict Result C *Initialize*: *i* ← 0, *P*(*N*) ← 0 **for** each *i* ∈ [1, N] do  *Normalized Data*: *I_i_* ← *BN*(*X_i_*)  *Extract spatial feature, filter size of*(1 × 30): *Cl_i_* ← *ReLU*(*BN*((*I_i_* · *W_s_* + *b_s_*))  *Pooling, apply with stride*(2 × 1): *M_i_* ← *maxpooling* (*C*1*_i_*)  *Extract temporal feature, filter size of*(20 × 1): *C*2*_i_* ← *ReLU*(*BN*((*M_i_* · *W_s_* + *b_s_*))  *Fully connected:*   F1*_i_* ← *fully connected*(*C*2*_i_*), *F*l*_i_* ← *Softmax*(*F*l*_i_*)   F2*_i_* ← *fully connected*(*F*2*_i_*)   P(*i*) ← F2*_i_*[1]**end for**C ← max(P)


## 3. Experiment and Result

### 3.1. Subjects

In this study, 10 healthy volunteers (5 males and 5 females) participated in our experiment after signing informed consent. The age of the subjects ranged from 22 to 27 years (mean age 23.8 years); SD 1.16 years. All of the subjects had normal or corrected-to-normal vision. They all had experience in playing the common Gomoku and had never used a BCI system before.

### 3.2. Experiment and Result

Prior to the experiment, the subjects were first instructed to read the participant information instructions to ensure that they understood the scene and purpose of the BCI game. We used a laptop with a bright 15.4 Liquid Crystal Display (LCD) screen with a 60 Hz refresh rate and 1080-pixel resolution. After ensuring that the subjects were in good condition, the experiment was conducted at about 10:00 a.m. In addition, a brief familiarization session was conducted before the experiment to ensure that subjects could adapt to the platform environment. The total experiment consisted of two parts: a training session and an online session (experiment I and experiment II). During the experiment, the subjects sat in a chair 0.5 m in front of the computer screen running the game in a quiet and comfortable room. At the same time, they were asked to relax and try not to produce unnecessary physical activity.

#### 3.2.1. Training Session

To prevent the constantly changing GUI from affecting the user’s attention, we only used the first-level paradigm to collect training data. In this session, the subjects used the first-level interface to continuously collect 40 items of training data. The subjects were asked to focus on the target character button on the stimulation panel displayed in the textbox and mentally count the number of flashes during the training session. The collected training data were preprocessed and then entered into the SBCNN algorithm for training.

#### 3.2.2. Experiment I

In experiment I, the subjects selected the target coordinates that were randomly given by the system and displayed in the textbox at the upper right of the GUI. Each subject was to perform 20 complete trials in experiment I, each of which consisted of two sub-trials. During the first sub-trial, the subject chose the region where the target coordinates were located in the first-level interface. Then the subjects chose the specific position in the second-level interface during the second sub-trial. In each sub-trial, all the valid characters in the interface randomly flashed in one round. After 10 rounds, the most possible target was selected based on real-time signal processing and presented in the textbox as feedback. Thus, the system outputted one coordinate in a complete trial through two selections. If the target coordinates were correctly selected, the pieces were placed on the board; otherwise not.

**Figure 4:**
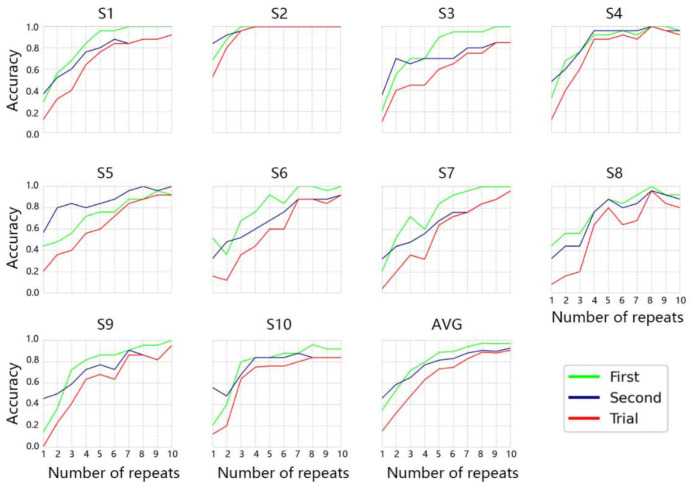
Each subject’s off-line accuracies and all subjects’ average accuracies in different repeats in experiment I. The vertical axis corresponds to the accuracy rate, and the horizontal axis corresponds to the number of repeats. The color curves represent classification accuracy rates in the first sub-trial, the second sub-trial, and the complete trial.

Table 2 lists the online target recognition accuracy rate of the first sub-trial, the second sub-trial, and the complete trial for the 10 subjects in experiment I. The online accuracy of all subjects in the first sub-trial was between 92% and 100%, with an average accuracy of 97.2%. The online accuracy of the second sub-trial was between 84% and 100%, and the average accuracy was 92.9%. The online accuracy of the complete trial is between 80% and 100%, with an average accuracy of 90.7%. There was no significant gender difference in the experiment through analysis of variance (first sub-trial: F(1,8) = 0.1, *p* = 0.7599; second sub-trial: F(1,8) = 0.58, *p* = 0.4695; complete sub-trial: F(1,8) = 0.87, *p* = 0.3792; ANOVA). These results confirm that P300 signals can be well evoked and recognized by our proposed system. Figure 4 presents the off-line average recognition accuracy rate and each subject’s accuracy rate in the first sub-trial, the second sub-trial, and the trial on different repeats. The different color lines in the figure record the results in the first sub-trial, the second sub-trial and the complete trial on each repeat. The average accuracy of all subjects reached 82% in the seventh repeat and finally reached 90.7% in the tenth repeat in the complete trial. In the first sub-trial, the average accuracy reached 80% in the fourth repeat and finally reached 97.2% in the tenth repeat. In the second sub-trial, the average recognition accuracy reached 80% in the fifth repeat and finally reached 92.9%. Each subject except S3 achieved an accuracy higher than 80% after the eighth repeat in the complete trial. In the first sub-trial, all subjects except S5 reached an accuracy higher than 80% after the fourth repeat. All subjects except S7 in the second sub-trials reached an accuracy rate of 80% or more after the seventh repeat. These results indicate that the proposed SBCNN algorithm could classify the P300 signal well and that our proposed system is stable and feasible.

**Figure 5:**
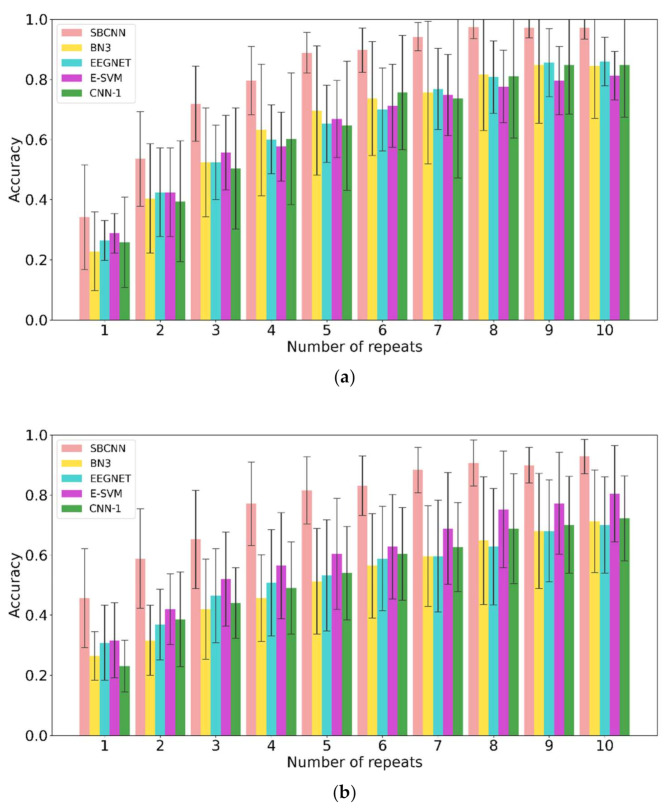

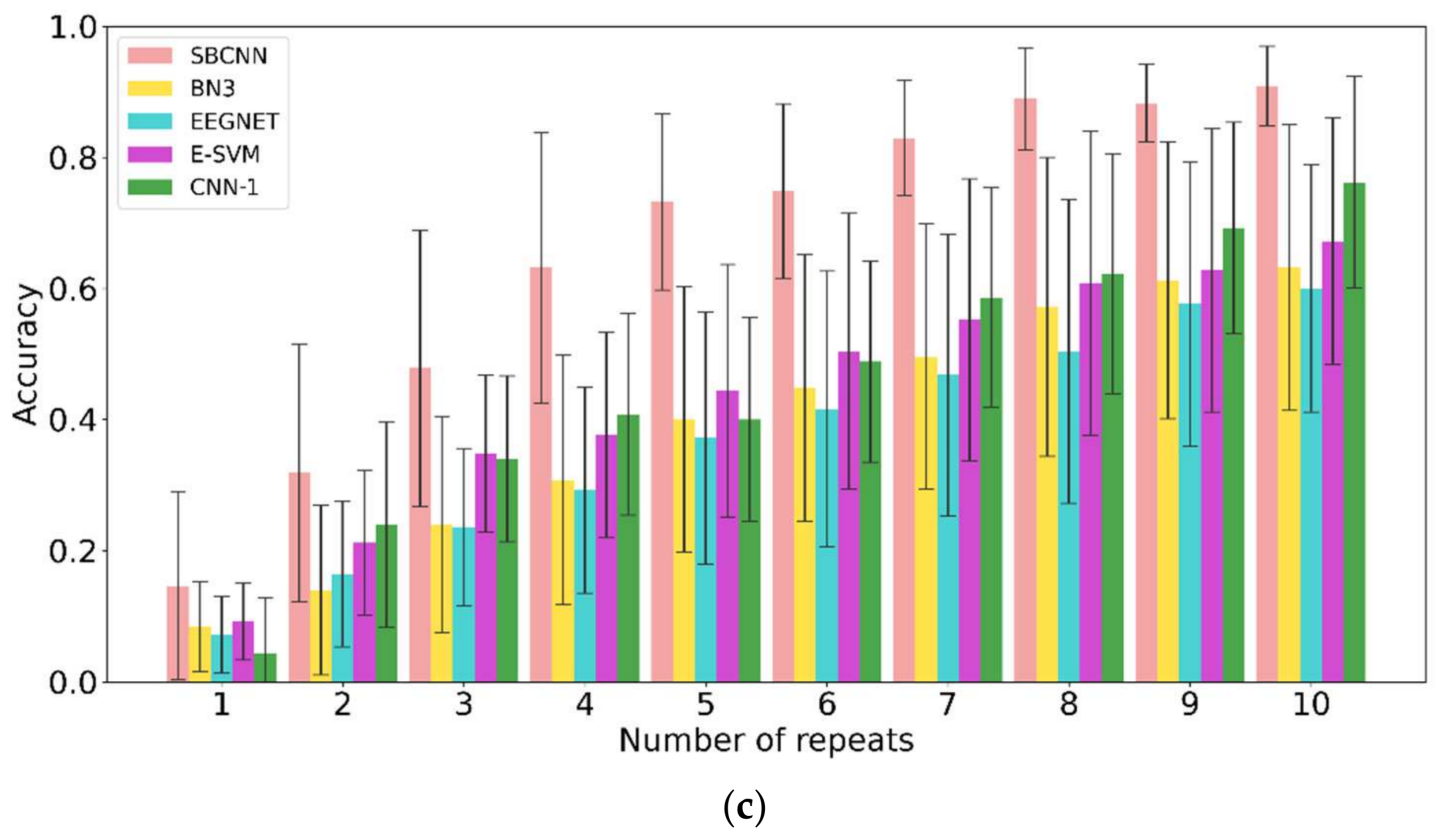
The mean ± standard classification accuracies of all 10 subjects in different repeats during experiment I: (**a**) in the first sub-trial, (**b**) in the second sub-trial, and (**c**) in the complete trial.

Figure 5 shows the average accuracy comparison between our proposed SBCNN algorithm and other classification methods in the literature, including CNN algorithms BN3 [30], EEGNET [41], CNN-1 [29] and E-SVM [42]. In the complete trial, the average accuracy of the SBCNN was higher than that of the other four algorithms from the first repeat and finally reached an accuracy of 90.7% in the tenth repeat. A repeated measures analysis of variance (ANOVA) was applied in the accuracy comparison in the tenth repeat between the proposed SBCNN and the other four algorithms. The result shows that the average accuracy of the SBCNN is significantly higher than that of the other four algorithms in a complete trial (F(4,45) = 4.65, *p* < 0.005, Bonferroni correction). Furthermore, after the fifth repeat, the standard deviation of the accuracy using SBCNN was lower than that of the other four algorithms. In the first sub-trial, for all subjects, from the first repeat, the average accuracy of the proposed algorithm was higher than that of the other algorithms. In the tenth repeat, we achieved an average accuracy rate of 96.4%, which is not statistically different from that of other algorithms (F(4,45) = 2.14, *p* = 0.0908, Bonferroni correction). Moreover, from the second repeat, the standard deviation of the average accuracy of SBCNN was smaller than that of the other four algorithms. In the second sub-trial, the average accuracy of SBCNN for all subjects finally reached an accuracy rate of 92.9% in the tenth repeat and was significantly higher than that of the other four algorithms (F(4,45) = 4.36, *p* < 0.005, Bonferroni correction). In addition, the standard deviation of the average SBCNN accuracy was less than that of the other four algorithms.

#### 3.2.3. Experiment II

To verify the feasibility of the proposed approach for the MindGomoku, experiment II was conducted. The online session was used to study the real-environment performance of the subjects’ approach to playing the MindGomoku. The subjects were required to play the MindGomoku with the end goal of winning the game. There are 5 s breaks between two successive trials, and the interval between two sub-trials is still 2.5 s. This means that after each coordinate is selected, the subject has 5 s to consider the next target coordinate. During experiment II, if the selection in the first sub-trial was wrong, the subject could choose R in the second sub-trial to return to the first-level interface and reselect. In this case, this trial would be recorded as an invalid trial.

In this paper, we also evaluated some online statistical results in experiment II, such as the number of complete trials, the number of valid pieces, the control accuracy, the total time of experiment II, the time per trial, and the result of the game, as shown in Table 3. The number of valid pieces is equal to the total number of complete trials minus the number of invalid trials. The accuracy rate is obtained by dividing the number of valid trials by the total number of trials. The total time represents the duration of the entire experiment II. It can be seen from Table 3 that the online average accuracy of the 10 subjects was as high as 97% using the proposed system in the actual game during experiment II. The average time of playing the game for all subjects was 13.73 min (the longest was 16.46 min), and four of them finally won in the human–computer battle.

## 4. Discussion and Conclusions

In this paper, we propose a P300 BCI game named the MindGomoku and a game interaction paradigm based on the characteristics of the MindGomoku. Moreover, this study introduces a P300 detecting algorithm based on Bayesian deep learning. This system can handle EEG signal processing and feedback in real time. Online and off-line experiments were conducted on 10 subjects with normal cognitive function for evaluating the performance of the proposed BCI system. The experimental results show that the mean game control accuracy achieved is 90.7% and the averaged game duration for all users is above 11 min. Therefore, these results also demonstrate the stability and effectiveness of the proposed algorithm and system.

Prior to this study, researchers have proposed several BCI games. Finke et al. proposed the P300-based MindGame, and the classification rate of a single trial during the online operation reached 66% [43]. Bonnet et al. created a multiuser videogame called BrainArena, in which two users can play a simple football game by means of two BCIs. The mean classification accuracy in single-player mode was 71.25% and that in two-player mode was 73.9% [14]. Wang et al. combined MI and SSVEP to generate multiple commands to jointly control Tetris. The classification accuracy of MI and SSVEP reached 87.01% and 90.26, respectively [10]. Our proposed BCI game MindGomoku does not require users to send commands continuously and quickly. Thus, sufficient buffer time can be provided for users to avoid discomfort and interference caused by long-term continuous visual stimulation, which may lead to poor user experience. Compared with other games, several characteristics of our game itself, such as turn-based and invalid operation at the same coordinate, can help the player avoid watching the stimulation panel for a long time and reduces the probability of visual fatigue. The online results of experiment II in Table 3 show that the accuracy of all subjects in the actual control can be higher than 85% and the average accuracy can reach 90.7%. This indicates that all subjects could use this system to play the MindGomoku for human–computer battles and the system runs stably for an average of 13 min. In this method, we use a dynamic flashing sequence to shorten the flashing time, which is generated by removing the valid buttons representing the occupied positions from all buttons.

To prevent a playing from paying the MindGomoku too slowly, we propose a game interactive paradigm that uses the GI-SS method to reduce stimulus interference and improve speed. In this method, a dynamic flashing sequence is applied to shorten the flashing time. To verify the performance of the GI-SS method, we respectively calculated the time per trial in experiments I and II. In experiment I, the time per trial for each subject was fixed, which was 30.2 s. As shown in Table 3, the time per trial for each subject is lower than 30.2 s. This indicates that GI-SS improves the timeliness of the system. However, as the ISI in the system is only 40 ms, a too-short stimulation sequence will cause severe repeated blindness [44,45]. In addition, GI-SS is just applied in the second-level interface because it is difficult for a region to be completely occupied, represented by a character button in the first-level interface. In short, system efficiency can be improved by reducing the stimulus sequence and the game interaction paradigm optimizes speed while ensuring accuracy.

Figure 4 presents the off-line average recognition accuracy rate and each subject’s accuracy rate in the first sub-trial, the second sub-trial, and the trial in different repeats. As shown in Figure 4, the average accuracy of the complete trial is lower than the accuracy of the first and second sub-trials. Because only the first sub-trial and the second sub-trial are correct, the complete experiment is correct, for all subjects except S5, as well as the average results of 10 subjects. The accuracy in the first sub-trial is higher than that in the second sub-trial. In addition, the average accuracy of all 10 subjects in the first sub-trial is higher than that in the second sub-trial. These findings may relate to task difficulty and fatigue. It is necessary for the P300 BCI to run many repeats to distinguish target and non-target stimuli that will cause user fatigue. In this study, the second sub-trial was always carried out after the first sub-trial. User fatigue may have caused the accuracy in the second sub-trial to be lower than that in the first sub-trial. In addition, in the first sub-trial, the subjects needed to choose a target character from 25 characters and in the second sub-trial from 10 optional characters. According to a previous report, the P300 amplitude increases as the probability of event-related stimuli decreases [46]. The difficulty of the task will also affect P300. If the task is difficult, the P300 wave is obvious, and vice versa [47]. In this study, the difficulty in the first sub-trial was higher than that in the second sub-trial. These reasons ultimately led to the above results.

**Figure 6:**
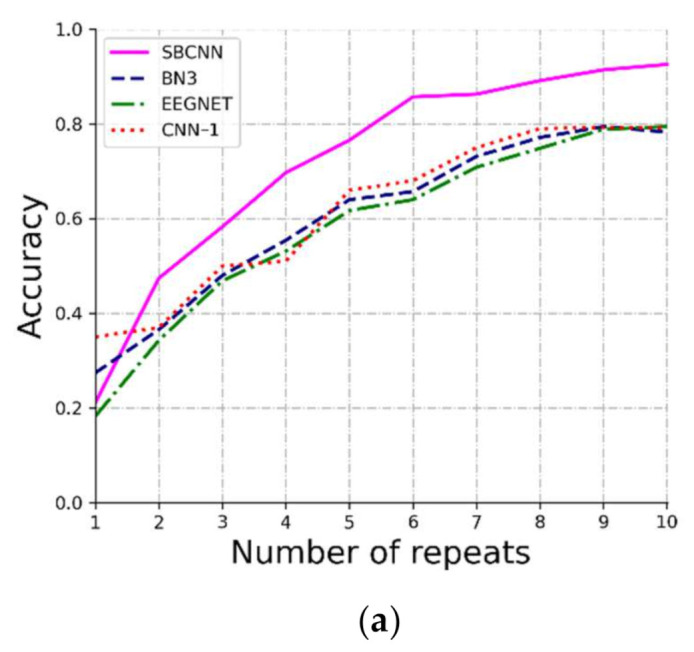

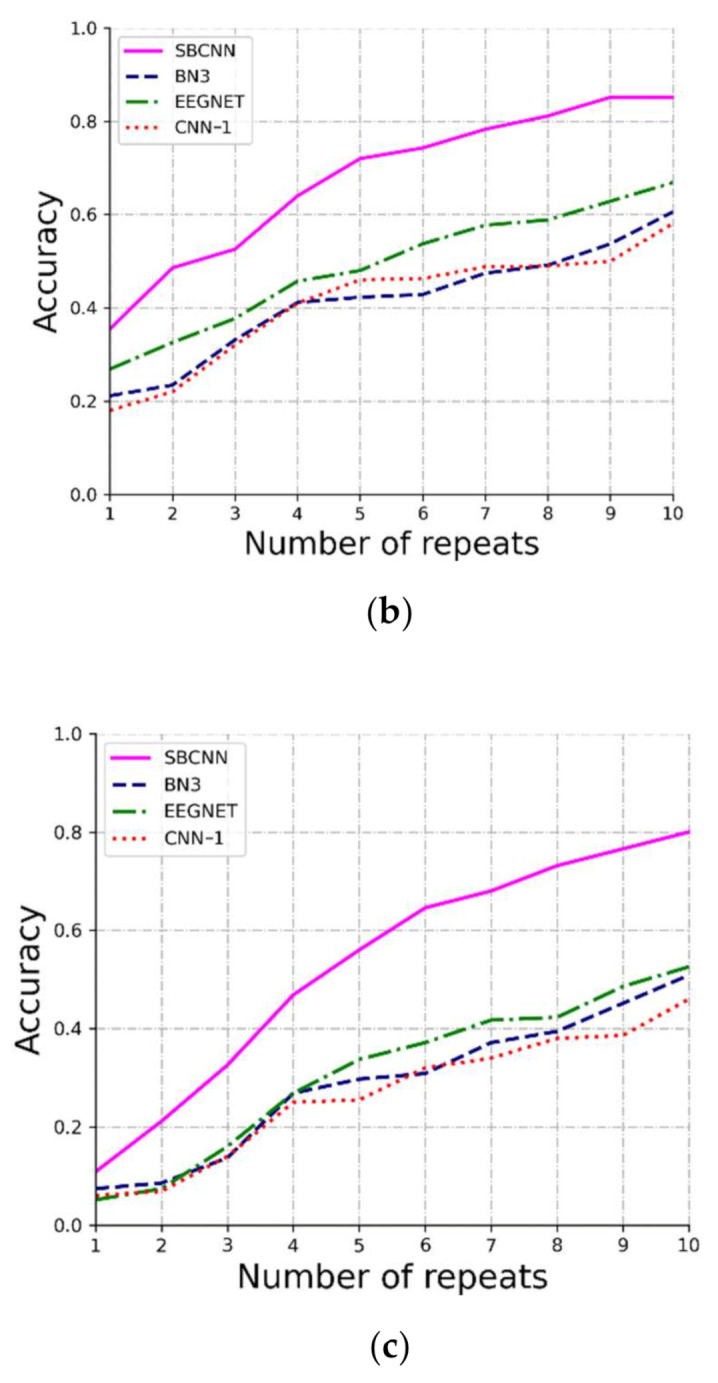
The average target recognition accuracy of all 10 subjects in different repeats trained on half training sets. The color curves represent classification accuracy rates in the SBCNN, BN3, EEGNET and CNN-1. (**a**) in the first sub-trial, (**b**) in the second sub-trial, and (**c**) in the complete trial.

Deep learning has achieved excellent results on large data sets in many research fields. However, in BCI applications, it is very difficult to obtain large amounts of data. In this paper, we propose a novel SBCNN algorithm based on a Bayesian convolutional neural network (BCNN) to detect a P300 signal. The BCNN offers good robustness to overfitting on small data by placing a probability distribution over the CNN kernels [39,48]. As shown in Figure 5, for the training set used in our experiment, the SBCNN had higher accuracy than the traditional algorithms and other CNN algorithms. In addition, the standard deviation of accuracies using the proposed algorithm in the first sub-trial, the second sub-trial, and the complete trial from the fifth repeat was lower than that using the other four algorithms. This also reveals that our SBCNN model is more stable than the other four methods. To further verify the superiority of the SBCNN algorithm on small data sets, we used half of the training sets in experiment I to train the classifier. The comparison results with three deep learning algorithms are shown in Figure 6. The different color lines in the figure record the results of all subjects that used different methods in each repeat. The accuracy of the SBCNN was obviously higher than that of the other methods from the second repeat in the first sub-trial, the second sub-trial, and the complete trial. Furthermore, all four algorithms worked better in the first sub-trial than in the second sub-trial. It may be due to the larger number of samples in the first sub-trial. In addition, the accuracy of SBCNN could reach as high as 80% in the complete trial. These experimental results show that the SBCNN is superior to the other two algorithms in small training sets and can achieve good results with small samples under the proposed paradigm.

This paper proposes an online BCI game based on a novel simplified Bayesian convolutional neural network, which can achieve good accuracy on a small training set and provide effective control of the game. Compared with some arcade games, the response time of the MindGomoku does not directly affect the progress of the game, and the turn-based mechanism avoids long-lasting visual stimulation. In the traditional the MindGomoku, the board coordinates of board games cannot be repeatedly selected. Therefore, in the proposed MindGomoku, we introduce the GI-SS method to reduce the control difficulty and improve the user experience. Online and off-line results show that our proposed MindGomoku is very stable in game function and operation accuracy. The motivation of this study is not only to prove the successful application of a P300-based BCI in computer games but also to prove the feasibility of turning disadvantages into advantages by combining gameplay and the characteristics of P300 BCI instructions. Finally, our experiments are currently only validated on healthy subjects. In the future, we will conduct experiments on subjects with disabilities. In addition, our experiments and data processing did not involve cross-subjects. Our next step will be to study a brain–computer interface system based on transfer learning.

## Figures and Tables

**Table 1:** The summary of related works on brain–computer interface (BCI) games.

BCI Game Studies	Subjects	Electrodes	Modality	Online Result (for N Subjects)
Martinez et al. [13]	*N* = 5	6	Steady-state visually evoked potential (SSVEP)	96.5% (medium frequency) 93% (low frequency)
Martišius et al. [15]	*N* = 2	4	SSVEP	78.2% (linear discriminant analysis (LDA)) 79.3% (support vector machine (SVM), linear kernel)) 80.5% (SVM, radial basis function kernel)
Bonnet et al. [14]	*N* = 20	8	Motor imagery (MI)	71.25% (single-player mode) 73.9% (two-player mode)
Wang et al. [10]	*N* = 10	20	SSVEP MI	90.26% 87.01%
Finke et al. [16]	*N* = 11	NA	P300	66%
Angeloni et al. [17]	*N* = 5	8	P300	88.47%

**Table 2:** The online accuracy rate of the first sub-trial, the second sub-trial, and the complete trial for all 10 subjects in experiment I.

Subject	S1	S2	S3	S4	S5	S6	S7	S8	S9	S10	Avg.
Gender	M	F	M	M	F	F	M	M	F	F	/
First sub-trial	100%	100%	100%	96%	92%	100%	100%	92%	100%	92%	97.2%
Second sub-trial	92%	100%	85%	96%	100%	92%	96%	88%	96%	84%	92.9%
Complete trial	92%	100%	85%	92%	90%	92%	96%	80%	96%	84%	90.7%

**Table 3:** Each subject’s online results in experiment II.

Subject	Trials	Valid Trials	Accuracy (%)	Total Time (min)	Time per Trial (s)	Win the Game
S1	18	14	94.4	11.8	25.3	Yes
S2	18	18	100	12.83	28.6	No
S3	17	14	100	11.61	26.9	No
S4	22	18	88.9	16.08	29.8	No
S5	20	20	100	13.1	25.3	No
S6	26	25	100	16.46	24.0	Yes
S7	21	19	100	13.11	23.5	No
S8	22	19	95.4	13.43	22.7	Yes
S9	26	24	100	15.65	22.1	No
S10	21	20	100	13.23	23.8	Yes
Avg	21.1	19.1	97.8	13.73	22.9	/

## Data Availability

The data used to support the findings of this study are available from the corresponding author upon request.
